# Satisfaction with remote consultations in primary care during COVID-19: a population survey of UK adults

**DOI:** 10.3399/BJGP.2023.0092

**Published:** 2024-01-23

**Authors:** Kate J Lifford, Detelina Grozeva, Rebecca Cannings-John, Harriet Quinn-Scoggins, Yvonne Moriarty, Ardiana Gjini, Mark Goddard, Julie Hepburn, Jacqueline Hughes, Graham Moore, Kirstie Osborne, Michael Robling, Julia Townson, Jo Waller, Victoria Whitelock, Katriina L Whitaker, Kate Brain

**Affiliations:** PRIME Centre Wales, Division of Population Medicine, School of Medicine, Cardiff University, Cardiff.; Centre for Trials Research, Cardiff University, Cardiff.; Centre for Trials Research, Cardiff University, Cardiff.; PRIME Centre Wales, Division of Population Medicine, School of Medicine, Cardiff University, Cardiff.; Centre for Trials Research, Cardiff University, Cardiff.; Public Health Wales; senior lecturer, Cardiff University, Cardiff.; Centre for Trials Research, Cardiff University, Cardiff.; Public Involvement Community, Health and Care Research Wales Support Centre, Cardiff.; Centre for Trials Research, Cardiff University, Cardiff.; DECIPHer (Centre for Development, Evaluation, Complexity and Implementation in Public Health Improvement), School of Social Sciences, Cardiff University, Cardiff.; Social & Behavioural Research, Cancer Research UK, London.; Centre for Trials Research, Cardiff University, Cardiff.; Centre for Trials Research, Cardiff University, Cardiff.; Centre for Cancer Screening, Prevention and Early Diagnosis, Wolfson Institute of Population Health, Faculty of Medicine & Dentistry, Queen Mary University of London, London; reader of cancer screening & early diagnosis, School of Cancer and Pharmaceutical Sciences, King’s College London, London.; Social & Behavioural Research, Cancer Research UK, London.; School of Health Sciences, University of Surrey, Guildford.; Division of Population Medicine, School of Medicine, Cardiff University, Cardiff.

**Keywords:** demographic factors, general practice, primary health care, remote consultations, telemedicine, telehealth

## Abstract

**Background:**

Mode of access to primary care changed during the COVID-19 pandemic; remote consultations became more widespread. With remote consultations likely to continue in UK primary care, it is important to understand people’s perceptions of remote consultations and identify potential resulting inequalities.

**Aim:**

To assess satisfaction with remote GP consultations in the UK during the COVID-19 pandemic and identify demographic variation in satisfaction levels.

**Design and setting:**

A cross-sectional survey from the second phase of a large UK-based study, which was conducted during the COVID-19 pandemic.

**Method:**

In total, 1426 adults who self-reported having sought help from their doctor in the past 6 months completed an online questionnaire (February to March 2021). Items included satisfaction with remote consultations and demographic variables. Associations were analysed using multivariable regression.

**Results:**

A novel six-item scale of satisfaction with remote GP consultations had good psychometric properties. Participants with higher levels of education had significantly greater satisfaction with remote consultations than participants with mid-level qualifications (*B* = −0.82, 95% confidence interval [CI] = −1.41 to −0.23) or those with low or no qualifications (*B* = −1.65, 95% CI = −2.29 to −1.02). People living in Wales reported significantly higher satisfaction compared with those living in Scotland (*B* = −1.94, 95% CI = −3.11 to −0.78), although caution is warranted due to small group numbers.

**Conclusion:**

These findings can inform the use and adaptation of remote consultations in primary care. Adults with lower educational levels may need additional support to improve their experience and ensure equitable care via remote consultations.

## Introduction

Over 133 500 excess deaths occurred during the COVID-19 pandemic (March 2020 to December 2021) in England and Wales, with peaks of excess deaths occurring in April 2020 and January 2021.^1^ Not only was there increased workload for the NHS during the COVID-19 pandemic, the mode of delivery and therefore mode of access for patients changed, with the use of remote consultations in primary care becoming widespread.^2^^,^^3^ Prior to this, remote consultations were used but evidence to support them as an alternative, in terms of both effectiveness and patient experience, was somewhat limited.^4^^–^^6^

A review of pre-pandemic studies noted inequalities in use of remote primary care consultations.^7^ Women and younger people were more likely to use remote consultations, and people aged >85 years and non-immigrants were more likely to use telephone consultations. ^7^ There was no clear pattern of association between other demographic or socioeconomic factors and remote primary care consultation usage.^7^ Although usage does not directly inform us about satisfaction with remote consultation, it may indicate preferences in pre-pandemic times when there was a choice about face-to-face or remote consultations.

At least some elements of remote GP consulting will likely continue beyond the pandemic. It is therefore important to consider patients’ experiences of remote consulting along with potential inequalities that might be exacerbated. The digital divide — the inequitable distribution of technology — has been highlighted, and its negative impact on health inequalities further fuelled by the pandemic.^8^ The increased use of remote consultations may have a greater impact on particular subgroups, such as individuals with limited access to the relevant technology^3^^,^^9^ or with dementia.^10^ A rapid review of patients’ experiences of remote primary care consultations during the pandemic identified both advantages and disadvantages of remote consultations perceived by patients.^11^ Findings about satisfaction with and preferences for remote consultation differed between studies.^11^ Some studies showed positive associations between satisfaction with remote consultations and demographic factors such as younger age,^12^^,^^13^ being female,^14^^,^^15^ higher education,^16^ and better health status.^17^ In contrast, no association was reported for age,^15^^–^^18^ gender,^12^^,^^13^^,^^17^ education,^12^^,^^17^ occupation,^16^ income,^12^^,^^16^ deprivation,^15^ or current health.^12^

**Table:** How this fits in

Remote consultations became more widespread during the COVID-19 pandemic and continue to date. However, patterns of association between demographic characteristics and satisfaction with remote GP consultations during the pandemic were unclear. People with higher levels of educational qualification were found to have greater levels of satisfaction with remote GP consultations. Those with lower educational levels may benefit from further support with remote consultations.

Against this backdrop, it is important to understand people’s perceptions of remote consultations and identify potential inequalities. The present study therefore aimed to assess satisfaction with remote GP consultations in the UK population during the COVID-19 pandemic and explore demographic variation in satisfaction levels.

## Method

### Setting and participants

Data for the present study were collected as part of a UK-based population survey conducted during the COVID-19 pandemic.^19^ Participants were invited to take part in the wider study between August and September 2020 (phase one) and again between February and March 2021 (phase two).^20^ Two UK-based population samples were recruited to complete an online questionnaire in both phases. Recruitment for the two samples was carried out via HealthWise Wales (HWW; a register for potential research participants) and social media for the COVID-19 Cancer Attitudes and Behaviours Study (CABS), and Dynata (an online panel provider commissioned by Cancer Research UK [CRUK]) for the COVID-19 Cancer Awareness Measure (COVID-CAM) data.^19^^,^^20^ For the CABS sample, potentially under-represented groups were targeted by specific recruitment strategies.^19^ For the COVID-CAM sample, quotas were placed on several characteristics to recruit a nationally representative and ethnically diverse sample.^19^ Eligibility criteria included being aged ≥18 years, living in the UK, and able to speak English. Questions on remote GP consultation were only included in the second phase of the wider study.

### Measures

Data were recoded where appropriate to ensure that responses from both samples were comparable. Response options ‘prefer not to say’ and ‘not applicable’ were treated as missing.

Demographic information was collected in both surveys through a series of multiple-choice questions. This included gender, age, ethnic background, highest educational qualification, employment status, relationship status, disability, place of residence, and presence of health conditions. Age was collected directly in the CABS sample, but was computed for the COVID-CAM sample using date of birth combined into 10-year categories. Participants were asked whether they had a variety of specific health conditions that were combined into one variable identifying the total number of health conditions reported.

Satisfaction with remote GP consultations was measured using seven items (see Supplementary Table S1) that were adapted from a CRUK survey,^21^ or developed responsively with stakeholders (via public/patient experiences and researchers’ objectives) during the study and tested for acceptability with lay representatives.^19^^,^^22^ Each item had response options on a 4-point Likert scale from ‘strongly disagree’ to ‘strongly agree’, with the additional options of ‘prefer not to say’ and ‘not applicable’. In the CABS sample, participants were only asked these seven items if they self-reported having sought help for a range of possible cancer symptoms (including vague/non-specific symptoms such as feeling tired all the time) during the preceding 6 months. Participants in the COVID-CAM sample who self-reported having tried to contact their GP practice in the last 6 months were included in the sample for analysis. Participants in both samples were asked the extent to which they agreed with each statement if they had received advice from a GP or doctor remotely (for example, a video or telephone call) for a health concern in the last 6 months.

### Statistical analyses

Data were analysed using IBM SPSS Statistics (version 27) and StataSE (version 17). Descriptive statistics were used to characterise the individual and combined samples. Principal component analysis (PCA) with varimax rotation was used to identify the underlying factor structure of items measuring satisfaction with remote GP consultations. Items that loaded (>0.3) on the extracted components from PCA were examined for potential inclusion in the final measure and were selected based on conceptual content, strength of factor loadings, component plot, and communalities. PCAs were conducted for both individual and combined samples to examine similarity. Selected items were reverse scored where appropriate and summed to form a scale with higher values indicating greater satisfaction. The internal consistency of the factor-derived scale was assessed using Cronbach’s alpha coefficient.

Frequency distributions (accompanied by percentages) for items were examined for each sample (CABS/COVID-CAM) and then combined (see Supplementary Table S1 for combined data for each item by demographic characteristics). *t*-tests and analysis of variance (ANOVA) (followed by *post hoc* Tukey tests) were used to examine differences in mean satisfaction scores by demographic factors. Multivariable linear regression analysis was conducted, including variables that were statistically significantly associated with satisfaction in univariable analyses. Each independent variable in the multivariable regression was identified as categorical, with the reference category being the group with the highest mean satisfaction score.

## Results

### Participant characteristics

The sample was derived from 4978 people who responded in the second phase of the wider study (response rate from first phase sample: *n* = 4978/7543, 66.0%). Of these, 1426/4978 (28.6%) people self-reporting help-seeking from their doctor in the previous 6 months were included in the present study. Just over half of the participants were male (51.8%), and the majority of participants (92.6%) were of a White ethnic background (Table 1). Most participants were aged between 55 and 74 years (52.5%), with a further 16.1% aged between 45 and 54 years. Over 40% of the sample were employed and a further 42.7% were retired. Over a third (36.9%) were educated to degree level or higher, with another third (33.9%) having further or higher education but below degree level.

**Table 1.** Participant characteristics for the combined (*n* = 1426) and individual samples (CABS, *n* = 457; COVID-CAM, *n* = 969)

**Characteristic**	**Combined sample,** ***n* (%)**	**CABS sample,** ***n* (%)**	**COVID-CAM sample,** ***n* (%)**
**Gender**			
Male	738 (51.8)	253 (55.4)	485 (50.1)
Female	685 (48.0)	204 (44.6)	481 (49.6)
Other	3 (0.2)	0 (0.0)	3 (0.3)

**Age, years**			
18–24	23 (1.6)	1 (0.2)	22 (2.3)
25–34	118 (8.3)	16 (3.5)	102 (10.5)
35–44	149 (10.4)	25 (5.5)	124 (12.8)
45–54	229 (16.1)	48 (10.5)	181 (18.7)
55–64	286 (20.1)	86 (18.8)	200 (20.6)
65–74	462 (32.4)	197 (43.1)	265 (27.3)
≥75	138 (9.7)	70 (15.3)	68 (7.0)
Other/prefer not to say	7 (0.5)	0 (0.0)	7 (0.7)
Missing	14 (1.0)	14 (3.1)	0 (0.0)

**Ethnic group**			
White	1321 (92.6)	441 (96.5)	880 (90.8)
Mixed/multiple ethnic groups	23 (1.6)	7 (1.5)	16 (1.7)
Asian/Asian British	52 (3.6)	1 (0.2)	51 (5.3)
Black/African/Caribbean/Black British	14 (1.0)	1 (0.2)	13 (1.3)
Other ethnic group	13 (0.9)	4 (0.9)	9 (0.9)
Prefer not to say	3 (0.2)	3 (0.7)	0 (0.0)

**Highest educational qualification**			
Degree or higher degree	526 (36.9)	206 (45.1)	320 (33.0)
Higher education qualification below degree level	220 (15.4)	90 (19.7)	130 (13.4)
A levels or Highers	214 (15.0)	39 (8.5)	175 (18.1)
ONC/BTEC	49 (3.4)	16 (3.5)	33 (3.4)
O levels or GCSE equivalent (Grade A–C/9–4)	240 (16.8)	55 (12.0)	185 (19.1)
O levels or GCSE equivalent (Grade D–G/3–1)	70 (4.9)	6 (1.3)	64 (6.6)
Still studying	6 (0.4)	1 (0.2)	5 (0.5)
No formal qualifications	75 (5.3)	32 (7.0)	43 (4.4)
Other	19 (1.3)	8 (1.8)	11 (1.1)
Prefer not to say	7 (0.5)	4 (0.9)	3 (0.3)

**Occupational status**			
Employed full time	389 (27.3)	72 (15.8)	317 (32.7)
Employed part time	156 (10.9)	41 (9.0)	115 (11.9)
Self-employed	83 (5.8)	20 (4.4)	63 (6.5)
Retired	609 (42.7)	277 (60.6)	332 (34.3)
Unemployed	50 (3.5)	8 (1.8)	42 (4.3)
Full-time homemaker	45 (3.2)	5 (1.1)	40 (4.1)
Disabled/too ill to work	76 (5.3)	29 (6.3)	47 (4.9)
Still studying	14 (1.0)	4 (0.9)	10 (1.0)
Prefer not to say	4 (0.3)	1 (0.2)	3 (0.3)

**Relationship status**			
Married	797 (55.9)	270 (59.1)	527 (54.4)
In a relationship	172 (12.1)	50 (10.9)	122 (12.6)
Single/never married	214 (15.0)	39 (8.5)	175 (18.1)
Divorced or separated	158 (11.1)	56 (12.3)	102 (10.5)
Widowed	80 (5.6)	39 (8.5)	41 (4.2)
Prefer not to say	5 (0.4)	3 (0.7)	2 (0.2)

**Number of health problems^a^**			
None	797 (55.9)	211 (46.2)	586 (60.5)
1	358 (25.1)	121 (26.5)	237 (24.5)
2	136 (9.5)	56 (12.3)	80 (8.3)
3	72 (5.0)	29 (6.3)	43 (4.4)
4	35 (2.5)	18 (3.9)	17 (1.8)
5–9^b^	28 (2.0)	22 (4.8)	6 (0.6)

**Disability**			
No	1022 (71.7)	298 (65.2)	724 (74.7)
Yes	366 (25.7)	148 (32.4)	218 (22.5)
Don’t know	29 (2.0)	9 (2.0)	20 (2.1)
Prefer not to say	9 (0.6)	2 (0.4)	7 (0.7)

**Country of residence**			
England	844 (59.2)	15 (3.3)	829 (85.6)
Wales	480 (33.7)	440 (96.3)	40 (4.1)
Scotland	73 (5.1)	2 (0.4)	71 (7.3)
Northern Ireland	21 (1.5)	0 (0.0)	21 (2.2)
Prefer not to say	8 (0.6)	0 (0.0)	8 (0.8)

*BTEC = Business and Technology Education Council. CABS = COVID-19 Cancer Attitudes and Behaviours Study. COVID-CAM = COVID-19 Cancer Awareness Measure. ONC = Ordinary National Certificate.*

a *Participants were given a list: arthritis, cancer, circulation problems, chest problems, depression, diabetes, heart problems, high blood pressure, kidney problems, stroke, and/or other.*

b *Data combined for ease of presentation.*

### PCA of satisfaction with remote GP consulting items

The results of PCA indicated an initial two-component solution with eigenvalues >1 (Kaiser’s criterion) accounting for 66.6% of the total variance (component 1: 51.2%, component 2: 15.4%). After varimax rotation, six out of seven items loaded (>0.3) onto component 1, two of which loaded onto both components (>0.3) but primarily onto component 1 (see Supplementary Table S2). Examination of the component plot showed that the only item that loaded exclusively (>0.3) onto component 2 (*‘In the future I would like to be offered the choice of a face-to-face consultation or remote consultation’*) appeared distinct from the others. Removing this item improved the internal consistency (α = 0.855; *n* = 1147 ‘complete cases’) and PCA showed that 58.4% of the total variance was explained by the one component solution (Table 2). The six remaining items fitted reasonably well together as a measure of satisfaction based on consideration of the factor loadings (all >0.6) and communalities (all but one >0.5) as well as conceptual issues. Results were similar when conducting PCA in the two individual samples (see Supplementary Tables S3–S6). Items were summed (reverse scoring where appropriate) to create a six-item satisfaction with remote GP consultations scale, with a total possible score range of 6 to 24 (higher scores indicating higher satisfaction). The scale was approximately normally distributed with a mean of 15.4 (standard deviation [SD] 4.29; range 6–24). Mean satisfaction scores were similar in the two individual samples (CABS mean 15.86, SD 4.23; COVID-CAM mean 15.22, SD 4.30).

**Table 2.** Final PCA of satisfaction with remote GP consulting items (*n* = 1147)

**Item**	**Component 1 factor loadings**	**Communalities**
Remote GP consultation allowed my health concerns to be adequately addressed	0.839	0.704
Remote GP consultations are more convenient for me compared with attending face to face	0.823	0.677
I feel comfortable discussing my health concerns via remote GP consultation	0.788	0.621
Remote GP consultations make me feel safer from coronavirus compared with attending face to face	0.739	0.546
I do not want remote GP consultations to continue after COVID-19	−0.733	0.538
I am concerned that remote GP consultations may result in the wrong decision being made about my care	−0.644	0.415

*PCA = principal component analysis.*

### Associations between satisfaction with remote GP consultations and demographic factors

Satisfaction with remote GP consultations was statistically significantly associated with age (*P* = 0.002), highest educational qualification (*P*<0.001), occupational status (*P*<0.001), and country of residence (*P* = 0.038) (Table 3). Specifically, *post hoc* tests showed that those aged 35–44 years were more satisfied with remote GP consultations than those who were aged 65–74 years (*P* = 0.005). Satisfaction with remote GP consultations increased with increasing level of education (all *P*<0.03). Those who were employed were more satisfied with remote GP consultations than those who were retired (*P*<0.001). Participants living in Wales reported greater satisfaction than those living in Scotland (*P* = 0.02).

**Table 3.** Univariable and multivariable associations between satisfaction with remote GP consultations and demographic factors

**Characteristic**	***n***	**Mean (SD) of satisfaction score^a^**	**Univariable analyses**		**Multivariable analyses**		

			**Test statistic (df)**	***P*-value**	***B***	**95% CI**	**Overall *P*-value**
**Gender**			*t*_(1143)_–0.86	0.389			
Male	591	15.3 (4.2)			*—*	*—*	*—*
Female	554	15.5 (4.3)			*—*	*—*	*—*

**Age, years**			F_(6, 206.1)_ 3.51^b^	0.002			0.31
18–24	21	16.2 (3.6)			−0.13	−2.17 to 1.90	
25–34	100	16.1 (4.0)			−0.31	−1.41 to 0.80	
35–44	125	16.4 (3.9)			Reference		
45–54	175	15.4 (4.4)			−0.77	−1.74 to 0.21	
55–64	226	15.7 (4.3)			−0.55	−1.50 to 0.40	
65–74	377	14.8 (4.4)			−1.40	−2.53 to −0.27	
≥75	107	15.0 (3.9)			−1.23	−2.58 to 0.12	

**Ethnic group**			*t*_(1142)_ −1.61	0.108			
White	1062	15.4 (4.3)			*—*	*—*	*—*
Ethnic minorities^c^	82	16.1 (4.3)			*—*	*—*	*—*

**Highest educational qualification**			F_(2, 1122)_ 17.42	<0.001			<0.001
Degree or higher degree	434	16.3 (4.1)			Reference		
Mid-level qualifications^d^	388	15.3 (4.2)			−0.82	−1.41 to −0.23	
No or low qualifications	303	14.4 (4.4)			−1.65	−2.29 to −1.02	

**Occupational status**			F_(2, 1141)_ 7.66	<0.001			0.50
Employed	506	16.0 (4.2)			Reference		
Retired	489	14.9 (4.3)			−0.27	−1.12 to 0.59	
Not employed^e^	149	15.1 (4.5)			−0.45	−1.26 to 0.35	

**Relationship status**			F_(2, 1142)_ 0.64	0.525			
Married/in a relationship	801	15.4 (4.3)			*—*	*—*	*—*
Single/never married	164	15.6 (4.1)			*—*	*—*	*—*
Divorced/separated/widowed	180	15.1 (4.5)			*—*	*—*	*—*

**Health problems^f^**			*t*_(1145)_ 1.61	0.108			
No health problems	632	15.6 (4.3)			*—*	*—*	*—*
At least one health problem	515	15.2 (4.3)			*—*	*—*	*—*

**Disability**			*t*_(469.8)_ 1.14	0.254			
No	832	15.5 (4.2)			*—*	*—*	*—*
Yes	288	15.2 (4.5)			*—*	*—*	*—*

**Country of residence**			F_(3, 1136)_ 2.86	0.038			0.009
England	684	15.4 (4.2)			−0.52	−1.09 to 0.05	
Wales	377	15.7 (4.3)			Reference		
Scotland	59	14.0 (3.9)			−1.94	−3.11 to −0.78	
Northern Ireland	20	15.4 (5.1)			−0.90	−2.81 to 1.01	

*BTEC = Business and Technology Education Council. df = degrees of freedom. ONC = Ordinary National Certificate. SD = standard deviation.*

a *Total possible score range of 6 to 24 (higher score indicating higher satisfaction).*

b *Asymptotically F distributed. Welch test reported because of heterogeneity of variances.*

c *Includes any ‘mixed/multiple ethnic groups’, ‘Asian/Asian British’, ‘Black/African/Caribbean/Black British’, and ‘other ethnic group’.*

d *Includes ‘higher education qualification below degree level’, ‘ONC/BTEC’, and ‘A-levels or Highers’.*

e *Includes ‘unemployed’, ‘still studying’, ‘full- time home maker’, and ‘disabled/too ill to work’.*

f *Participants were given a list: arthritis, cancer, circulation problems, chest problems, depression, diabetes, heart problems, high blood pressure, kidney problems, stroke, and/or other.*

Multivariable analysis including age, education, occupation, and country of residence (Table 3) explained 5% of the variance in satisfaction (*F*_(13, 1086)_ = 4.759, *P*<0.001). When adjusting for the other factors, highest education and country of residence were significantly associated with satisfaction. Those educated to degree level or above had significantly higher satisfaction scores than those with mid-level qualifications (*P* = 0.006) and those with no or low-level qualifications (*P*<0.001). People residing in Wales had significantly higher satisfaction scores than those residing in Scotland (*P* = 0.001). Overall, age was not associated with satisfaction with remote GP consultations, although the initial difference in satisfaction between those aged 35–44 years and those aged 65–74 years (*P* = 0.02) was still evident.

## Discussion

### Summary

A UK population survey of satisfaction with remote GP consultations during the COVID-19 pandemic was conducted. The six-item satisfaction scale had good internal consistency and was approximately normally distributed. Higher educational level and residence in Wales (compared with Scotland) were associated with higher satisfaction.

### Strengths and limitations

This study was based on a large UK sample. While there was good representation from England and Wales, representation from Northern Ireland and Scotland was limited. The results pertaining to the country of residence should therefore be interpreted with caution. There was also limited representation of young adults (aged 18–24 years) and individuals from ethnic minority groups.

The authors acknowledge measurement limitations in the present study. First, the different methods of identifying participants within the two samples who had experienced remote GP consultations (that is, filtering based on help-seeking for self-reported symptoms versus for any health concern) may have influenced the results. It is possible that satisfaction with remote primary care encounters may vary depending on the nature of the health issue in question. However, the association between type of health problem and satisfaction was not examined in the present study.

Second, the satisfaction scale items refer generically to remote consultation, which may include synchronous (for example, telephone) and asynchronous (for example, email) modes. While this was intended to reflect the varied usage of remote consultations in UK primary care, the present study was not designed to assess potential differences in satisfaction by remote consulting mode.

Finally, satisfaction with remote GP consultations was not measured in the first phase of the wider study,^19^ thus changes in satisfaction during the pandemic could not be assessed. However, this study provides an important benchmark for levels of satisfaction in the UK population during the pandemic. The overall and subgroup satisfaction scores were close to the scale midpoint, suggesting potential for improvement for all groups.

### Comparison with existing literature

Consistent with the present findings, higher education was associated with higher satisfaction with telephone consultations for antenatal care provided during the pandemic.^16^ However, two other studies have not found an association between education and satisfaction with remote consultations.^12^^,^^17^ Both studies involved samples with high levels of education, suggesting there was insufficient variation to observe an effect. Two pre-pandemic studies from a review^7^ broadly supported an association between higher educational level and use of technology.^23^^,^^24^ This is also consistent with the digital divide.^8^ Technological capability and satisfaction with remote consultations may be interrelated, although this was not assessed within the present study. Those with higher levels of education may have both better access to and knowledge of using technology for remote consultations leading them to feel more confident in its use, resulting in higher levels of both use and satisfaction.

Studies exploring demographic variation in satisfaction with remote primary care consultations during the COVID-19 pandemic have used a variety of satisfaction measures, often using single items.^11^^–^^15^^,^^18^ Those which used multi-item measures of satisfaction do not appear to have included factors specific to COVID-19.^16^^,^^17^ Inclusion of pandemic-related items is important because they may reflect the impact of the context (that is, a pandemic) on how people perceive their satisfaction with remote consultations.

### Implications for research and practice

The authors developed a robust measure of satisfaction with remote GP consultations that would benefit from further psychometric testing (for example, test–retest reliability) and scale validation in different groups and settings. Testing the scale in settings using specific modes of remote consultation (for example, email, eConsults, and telephone) would be useful to both assess the psychometric properties of the generic scale items and explore associations with demographic characteristics. Two scale items were specific to the COVID-19 pandemic but could be adapted and tested in other healthcare contexts, for example, participants could be asked about feeling safe from catching communicable diseases.

It is imperative to understand how people, particularly those with lower levels of education, can be better supported in remote consultations to improve their satisfaction. Further research to understand the behavioural and social factors (for example, access to and usage of technology) underpinning the association with education is needed. UK-wide studies exploring the possible association between country of residence and satisfaction may be beneficial. If confirmed, it will be important to understand whether this reflects variation in health service provision in the devolved UK nations. Despite initial associations, age and occupational status were not overall significantly associated with satisfaction in the multivariable analyses, suggesting they were correlated with other factors. Multivariable exploration of the association with age in other samples will be useful, particularly given the mixed findings in previous studies.^12^^,^^13^^,^^15^^–^^18^ Given the limited representation of those from ethnic minority groups in the present study, further exploration of satisfaction with remote GP consultations by ethnic background would be useful. Only a small proportion of the variation in satisfaction (5%) was explained by demographic factors, suggesting further exploration of unmeasured factors is warranted. This may include factors such as the type and severity of the health problem and relationship with the clinician,^15^^,^^25^^–^^27^ as well as mode of remote consultation (for example, email, eConsults, and telephone).

The present findings can be used to inform the use and adaptation of remote consultations in primary care for particular subgroups of the population. Individuals with lower levels of education may need further support with remote consultations in primary care to improve their satisfaction or indeed be offered face-to-face consultations if a feasible alternative. This will be vital to ensure equitable satisfaction with consultations and to mitigate potential inequalities in access to primary healthcare services.

## Data

Information on the data underpinning the results presented here, including how to access them, can be found at the UK Data Service at https://doi.org/10.5255/UKDA-SN-855905.
